# Restricted Network Reconstruction from Time Series via Dempster–Shafer Evidence Theory

**DOI:** 10.3390/e28020148

**Published:** 2026-01-28

**Authors:** Cai Zhang, Yishu Xian, Xiao Yuan, Meizhu Li, Qi Zhang

**Affiliations:** 1 School of Science, Jiangsu University of Science and Technology, Zhenjiang 212100, China; zhangcai_just@163.com (C.Z.);; 2 School of Computer Science and Communication Engineering, Jiangsu University, Zhenjiang 212100, China; 3 Lorentz Institute for Theoretical Physics, Leiden University, P.O. Box 9504, 2300 RA Leiden, The Netherlands

**Keywords:** network reconstruction, Dempster–Shafer (DS) evidence theory, multi-source information fusion, complex networks

## Abstract

As a fundamental mathematical model for complex systems, complex networks describe interactions among social, infrastructural, and biological systems. However, the complete connection structure is often unobservable, making topology reconstruction from limited data—such as time series of unit states—a crucial challenge. To address network reconstruction under sparse local observations, this paper proposes a novel framework that integrates epidemic dynamics with Dempster–Shafer (DS) evidence theory. The core of our method lies in a two-level belief fusion process: (1) Intra-node fusion, which aggregates multiple independent SIR simulation results from a single seed node to generate robust local evidence represented as Basic Probability Assignments (BPAs), effectively quantifying uncertainty; (2) Inter-node fusion, which orthogonally combines BPAs from multiple seed nodes using DS theory to synthesize a globally consistent network topology. This dual-fusion design enables the framework to handle uncertainty and conflict inherent in sparse, stochastic observations. Extensive experiments demonstrate the effectiveness and robustness of the proposed approach. It achieves stable and high reconstruction accuracy on both a synthetic 16-node benchmark network and the real-world Zachary’s Karate Club network. Furthermore, the method scales successfully to four large-scale real-world networks, attaining an average accuracy of 0.85, thereby confirming its practical applicability across networks of different scales and densities.

## 1. Introduction

Accurately portraying network topology is a prerequisite for revealing system functions, predicting dynamic behavior, and implementing effective interventions. The concept of complex networks has wide applicability, encompassing systems that exhibit characteristics such as self-organization, small-world properties, or scale-free structure. Its core value lies in providing an abstract yet powerful characterization of highly interconnected real-world systems. However, this abstraction faces a fundamental challenge: in most practical scenarios, the complete connection structure of a system cannot be directly observed. Biologists cannot simultaneously measure all protein–protein interactions, sociologists cannot fully track the dynamic evolution of every social tie, and engineers cannot monitor each connection in a large-scale infrastructure network in real time. This lack of structural information places us in a cognitive dilemma of “seeing the nodes but missing the connections.” Therefore, complex network topology reconstruction—that is, inferring unknown connections from limited and indirect observational data—has become a fundamental and pressing research direction in network science. Solving this problem is of great theoretical importance and broad practical value. In biomedicine, precisely reconstructing gene regulatory networks helps elucidate disease mechanisms [1,2]. In social science, reconstructing implicit social networks offers deeper insight into information diffusion within populations [2,3]. In infrastructure protection, identifying critical connections is essential for safeguarding system security and mitigating inherent risks [4,5]. Nevertheless, network reconstruction faces multiple challenges. First, observational data are often incomplete, typically containing information from only a subset of nodes or a limited time period. Second, noise and measurement errors further increase reconstruction uncertainty [6,7]. Moreover, different network types possess distinct topological characteristics, demanding the development of more universal methods. Finally, computational complexity remains a key obstacle for large-scale network reconstruction. These challenges collectively define the core scientific problems in the field of complex network topology reconstruction and have driven numerous methodological innovations, including the present study.

Complex network reconstruction is an inverse problem: it aims to infer the interaction architecture between system components from limited observational data. Solving this inverse problem provides a powerful means to understand and interpret complex systems through the lens of network models. As summarized by network scientist Tiago P. Peixoto [7,8,9,10], complex network reconstruction essentially involves inferring inter-unit relationships based on available information, employing methodologies such as Bayesian principles, compressed sensing, causal analysis, and maximum entropy principles [11]. From an information processing perspective, network topology reconstruction can be viewed as extracting inter-component relationships by processing temporal state data of system units. When reconstruction relies on multi-source temporal data, the inference methodology must incorporate theories for integrating such heterogeneous information. Thus, introducing evidence theory from information fusion to process multi-source temporal node states presents a feasible approach for inferring node relationships. Over the past two decades, researchers have proposed various complex network reconstruction methods. Early approaches primarily relied on correlation-based analysis of node time-series states to construct network topology [12,13]. Subsequently, scientists developed transfer entropy methods based on mutual information for network reconstruction [14]. Concurrently, Granger causality-based methods were proposed to infer network structures through causal relationships between random variables [15,16]. Later, linearization techniques for nonlinear dynamical equations were introduced to address network reconstruction from nonlinear time-series data [17,18]. Chinese scholars further advanced the field by incorporating compressed sensing and LASSO statistical inference methods, effectively addressing reconstruction under noisy conditions and extending these approaches to time-varying nonlinear dynamical systems [19,20,21]. During the same period, probabilistic graphical models were applied to network reconstruction [22,23,24]. In recent years, causal inference methods have been widely adopted for reconstructing network structures from time-series data, and graph neural network models (e.g., GGN) have demonstrated strong potential by inferring unobservable network components while maintaining robustness to noise and missing information [25,26,27]. Additionally, reconstruction methods based on epidemic models, Ising models, and others have been proposed [28,29,30]. These general methods based on node time-series data have significantly enriched the methodological toolkit for complex network reconstruction. Overall, single-source data-driven reconstruction has made remarkable progress, with a variety of effective techniques developed for different data types such as node time series, node degrees, and edge weights. However, when confronted with multi-perspective observational data and the conflicts arising from multi-source temporal information, the reconstruction problem transforms into one of complex uncertainty processing. Therefore, it remains imperative to develop a new methodological framework to address this challenge.

To address the challenges of sparse node data and information uncertainty in complex network reconstruction, this paper proposes an innovative framework that integrates propagation dynamics with information fusion. Its core approach is to explore the network structure through controlled information propagation and achieve robust reconstruction via orthogonal fusion of multi-source temporal data. At the level of propagation dynamics, the SIR (Susceptible–Infectious–Recovered) epidemic model is used to generate temporal data. Simulating this process yields temporal node states containing connectivity information, a method suitable for local observation scenarios that only require a few nodes as propagation sources. At the level of information processing, multi-source information fusion is introduced to enhance reconstruction performance. Information fusion aims to integrate multi-source data, reduce uncertainty, and resolve conflicting evidence. Recent research highlights that the order and strategy of fusion can significantly impact the final outcome. For instance, in frameworks like Random Permutation Set Theory, leveraging additional information to guide the fusion process has been shown to substantially improve decision accuracy [31,32]. This insight motivates our approach to carefully design the fusion mechanism for the temporal data from multiple SIR simulations. This study treats multiple independent SIR simulations as distinct information sources and performs orthogonal fusion of temporal data based on evidence theory, thereby improving reconstruction accuracy and robustness. This framework provides a systematic solution for network reconstruction under local observation, particularly applicable to real-world scenarios with incomplete data and information uncertainty.

Effectively managing the inherent uncertainty and conflicting information in observational data is crucial for the inverse problem of complex network reconstruction. This paper employs Dempster–Shafer (D-S) evidence theory as its core mathematical framework precisely due to its unique strengths in uncertainty reasoning. Beyond providing a rigorous formalism for representing and quantifying uncertainty, evidence theory offers a powerful mechanism for fusing multi-source, heterogeneous information—making it particularly suitable for network reconstruction under partial observation. Compared to traditional probabilistic methods, evidence theory holds three significant advantages. First, it explicitly separates “uncertainty” from “ignorance” through the Basic Probability Assignment (BPA), which simultaneously captures the degree of support, opposition, and uncertainty regarding a proposition. Second, it requires less prior information and does not presuppose a complete probability distribution. Third, Dempster’s combination rule furnishes a mathematically sound method for evidence synthesis, enabling the coherent fusion of even conflicting evidence. These theoretical merits have sustained the widespread adoption of evidence theory over the past four decades, from early expert systems and military target recognition to contemporary applications in artificial intelligence, medical diagnosis, and risk assessment. Especially in multi-sensor information fusion, it has become a standard methodology for handling uncertain, incomplete, and conflicting data. Applying evidence theory to network reconstruction in this work entails three key steps:

Evidence Representation

The time-series data obtained from each SIR simulation are transformed into basic probability assignments (BPA). For any node pair (i,j), three basic probability values are calculated based on their state transition patterns: m({T}) represents the degree of belief supporting the existence of an edge, m({F}) represents the degree of belief supporting the absence of an edge, and m({T,F}) represents the degree of uncertainty. This representation fully preserves the uncertainty information in the raw data.

Evidence Fusion

Dempster’s combination rule is used to synthesize evidence from multiple SIR simulations. The fusion process is to integrate each piece of evidence orthogonally in order: first, the two pieces of evidence are orthogonalized, and then the fusion results are orthogonally fused with the third piece of evidence, and so on until all the evidence is orthogonally integrated. The conflict coefficient *k* will automatically adjust the weight of each piece of evidence throughout the integration process, thus reducing the impact of conflicting evidence on the final integration result.

Decision-Making

Evidence decision-making is based on the final result of orthogonal fusion, which considers not only the degree of support for connections between nodes but also the uncertainty and conflict inherent in the evidence. In our current implementation, a classical probabilistic transformation is applied to convert the fused Basic Probability Assignment (BPA) into a probability distribution for binary edge classification. It is noteworthy that the field of evidence theory is actively refining this critical transformation step. Recent advanced methods, which integrate belief entropy with structural representations to derive more accurate and interpretable probability distributions [33], represent the cutting edge in uncertainty-aware decision-making. While our framework effectively utilizes the core fusion strengths of D-S theory, it remains fully compatible with and can be directly enhanced by such sophisticated transformation techniques in future work.

The innovation of this work is reflected in three primary aspects. First, it pioneers the application of evidence theory to network reconstruction under local observation, establishing a novel methodological pathway. Second, it designs a complete, end-to-end reconstruction pipeline—from data generation to evidence formation and final decision-making—thereby providing an actionable general framework. Third, it constructs a rigorous mathematical foundation that ensures theoretical solidity throughout the process. Compared with existing approaches, the proposed evidence-theoretic method offers distinct advantages. It not only quantifies the uncertainty inherent in the reconstruction process into concrete numerical measures but also introduces a new mechanism for multi-source information integration, resulting in superior robustness when reconstructing networks from very few information sources. Consequently, our method is particularly well-suited for addressing common practical challenges such as local observation, data noise, and conflicting or uncertain information.

In summary, this paper proposes an innovative method for complex network topology reconstruction that integrates SIR epidemic dynamics with D-S evidence theory. The core of the method lies in leveraging the temporal state matrices generated by SIR propagation, coupled with a two-level evidence fusion process, to significantly improve reconstruction accuracy and robustness. The main contributions of this work can be summarized at the following three levels: Inter-node correlation based on state transition: This work proposes a criterion for inferring connectivity between nodes: if node *i* and node *j* exhibit an infectious-susceptible state transition between time *t* and t+1, a connection likely exists between them. By statistically aggregating the frequency of such state transitions, we quantify the likelihood of an edge. This approach captures causal propagation pathways in the network, thereby reflecting the underlying connectivity. Uncertainty handling of single-source temporal information: To address the conflict and uncertainty inherent in SIR propagation data from a single seed node, we introduce Dempster–Shafer (D-S) evidence theory to preprocess the initial correlation matrix. Through Basic Probability Assignment (BPA), we distinguish three possibilities—support for connection, opposition to connection, and uncertainty—effectively managing redundant and conflicting information among unrelated nodes. This step lays a high-quality evidential foundation for subsequent fusion. Collaborative fusion framework for multi-source evidence: The core contribution of this work is a multi-source evidence fusion framework. Treating the BPA results derived from SIR propagation starting from different seed nodes as independent bodies of evidence, we progressively integrate them using Dempster’s combination rule. This orthogonal fusion fully exploits the complementary nature of temporal information from distinct infection sources, substantially improving reconstruction accuracy and offering a novel, reliable solution for network reconstruction under local observation.

In summary, to address the critical challenge of network reconstruction under extreme local observation, this paper establishes an innovative paradigm that fundamentally integrates dynamical propagation simulation with evidential information fusion. This “Local Observation and Uncertainty Fusion” paradigm shifts the focus from traditional global-inference techniques to a framework capable of handling sparse data and inherent uncertainty. The remainder of this paper is structured as follows: Section 2 introduces the foundational theories, including complex networks, the SIR epidemic model, and D-S evidence theory. Section 3 elaborates on the proposed belief fusion-based reconstruction methodology, which embodies the new paradigm. Section 4 validates the necessity and effectiveness of the paradigm through systematic experiments and ablation studies on real-world networks under strict local-observation constraints. Finally, Section 5 concludes the paper and discusses future research directions enabled by this paradigm shift.

## 2. Preliminary

### 2.1. Complex Networks

Complex networks serve as a fundamental cross-disciplinary model for characterizing real-world systems. A classical description was provided: a network possessing some or all properties of self-organization, self-similarity, attractors, small-worldness, or scale-freeness. Broadly speaking, complex networks refer to large-scale networks composed of a vast number of nodes and non-trivial connections, whose topological structures exhibit statistical regularities. They are distinct from both entirely regular and completely random networks, providing an abstract representation of real systems such as social relationships, transportation links, and biological metabolism.

From a mathematical essence perspective, complex networks are quantitatively described using a Graph, denoted as G=(V,E). Here, $V=\{v_{1},v_{2},\ldots ,v_{N}\}$ is the set of nodes containing *N* elements, representing entities in a system (e.g., individuals in epidemic spread, users in a social network). $E=\{e_{ij}\}$ is the set of edges, where $e_{ij}=1$ if an interaction exists between nodes $v_{i}$ and $v_{j}$, and $e_{ij}=0$ otherwise, characterizing the relationships between entities.

The most fundamental tool for describing the graph structure is the Adjacency Matrix *A*. This matrix is an N×N square matrix whose elements $A_{ij}$ correspond one-to-one with the edges $e_{ij}$: in undirected networks $A_{ij}=A_{ji}$ (the matrix is symmetric), whereas in directed networks $A_{ij}$ and $A_{ji}$ are independent (representing connections in different directions). The core objective of network reconstruction is to accurately infer the values of the elements of this adjacency matrix *A* from limited observational data, thereby recovering the entire network’s topological structure.

The effectiveness of networks in characterizing complex systems lies in the rich information contained within their topological structures. By analyzing topological features such as clustering analysis, local centrality of nodes, and network entropy, we can gain deep insights into the macroscopic behaviors and underlying mechanisms of the system. In particular, the concept of entropy has been extended to understand the fundamental statistical physics of network ensembles, which are models for systems with heterogeneous interactions. Recent work has shown that network ensembles are inherently nonextensive, a property emerging from the freedom of nodes to connect, as revealed through the analysis of local entropy [34]. This provides a powerful framework (local entropy) for probing the origin of complexity in networked systems, differentiating it from constrained interaction models like the Ising model.

The topological properties of complex networks are key features that distinguish them from simple networks and can be categorized into the following three types:

#### 2.1.1. Definition 2.1 (Average Path Length)

Defined as the average length of the shortest paths between all pairs of nodes in the network, it reflects the efficiency of information or material transmission within the network. When the average path length increases logarithmically with the number of nodes, the network exhibits the small-world property. For instance, in the Karate Club network [35], any two members can typically be connected through an average of 2–3 intermediate nodes. This property enables the potential for ‘rapid spread’ in real systems, such as infectious diseases or information diffusion.

#### 2.1.2. Definition 2.2 (Clustering Coefficient)

This measures the closeness of connections between a node’s neighbors. The local clustering coefficient of a single node $v_{i}$ is $C_{i}=\frac{2E_{i}}{k_{i}\left(k_{i}-1\right)}$ (where $k_{i}$ is the degree of node $v_{i}$, and $E_{i}$ is the number of edges that actually exist between its $k_{i}$ neighbors). The network’s overall clustering coefficient is the average of all $C_{i}$. A high clustering coefficient implies the existence of ‘local clustering’ within the network. For example, in the Karate Club, the connection density among members within the same faction is significantly higher than that between factions. This characteristic directly influences local dynamics, such as the speed of epidemic outbreaks within small groups.

#### 2.1.3. Definition 2.3 (Degree Distribution)

This represents the probability that a randomly selected node has degree *k*, and it is a core indicator for distinguishing network types. Regular networks have a Delta function degree distribution (all nodes have the same degree), random networks follow a Poisson distribution (most nodes have degrees close to the average), while scale-free networks follow a power-law distribution $P\left(k\right)\propto k^{-\gamma }$ (with γ∈[2,3])—meaning a few nodes have very high degrees (called ‘hub’ nodes), while the majority of nodes have low degrees. Although the Karate Club network, due to its small size, does not exhibit a strict power-law distribution, the high connectivity of its core nodes (e.g., the club manager) shares structural similarities with the hub effect observed in scale-free networks.

**Figure 1:**
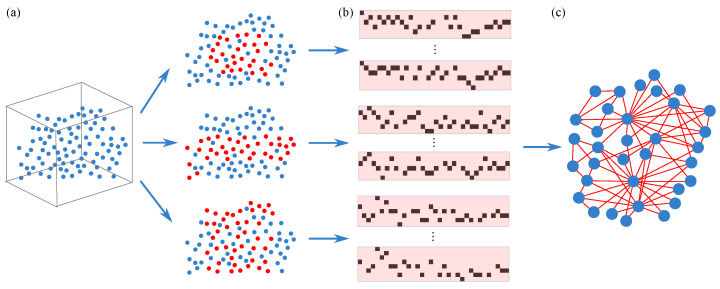
Framework for complex network topology reconstruction based on multi-source time-series information. (**a**) Real-world complex system and its multi-perspective observation sources; (**b**) Multi-source time-series data obtained from observations; (**c**) Reconstructed complex network topology via the proposed method.

Precisely because the complete topological structure of real-world complex networks is often unknown and difficult to observe directly—for instance, the inability to fully obtain all contact relationships between individuals during epidemic spread, or the challenge in directly recording implicit interactions between users in social networks—reconstructing the network topology from limited, indirect observational data has become a fundamental and critical scientific problem in network science. The research objective of this paper is to address this inverse problem: to establish a methodology capable of accurately inferring the underlying topological structure of associations between system units from multi-source temporal observation data of a complex system. This research will provide the theoretical foundation and a practical pathway for the subsequent network reconstruction based on belief fusion. The overall research framework is illustrated in Figure 1.

### 2.2. SIR Epidemic Model

The SIR (Susceptible–Infectious–Recovered) model is a classic epidemiological model proposed by Kermack and McKendrick in 1927 [36]. Its core principle involves dividing the study population into three mutually exclusive states and describing the transition rules between these states through dynamical equations. It remains a fundamental theoretical basis for constructing network propagation models. The state classifications and their biological meanings are as follows:

- Susceptible (S): Healthy individuals who are not infected but are at risk of infection and can be infected by infectious individuals.
- Infected (I): Individuals who have been infected with the virus possess the ability to transmit it and can spread the virus to susceptible individuals.
- Recovered (R): Individuals who have recovered from the infected state, gaining lifelong immunity. They are considered to have been removed from the follow-up transmission system.

#### 2.2.1. Dynamical Equations

Under the condition that the contact probability of all individuals is equal, the propagation process of the SIR model can be quantitatively described by the following system of differential equations:

(1) $$\begin{array}{c} \left\{\begin{array}{c} \frac{dS(t)}{dt}=-\frac{\beta S(t)I(t)}{N}\\ \frac{dI(t)}{dt}=\frac{\beta S(t)I(t)}{N}-\gamma I\left(t\right)\\ \frac{dR(t)}{dt}=\gamma I\left(t\right) \end{array}\right. \end{array}$$

Among them, S(t), I(t), and R(t) represent the number of susceptible, infected, and recovered at moment *t*, respectively. N=S(t)+I(t)+R(t) is the overall scale. The infection rate β indicates the average number of susceptible people that a single infected person can infect in a unit of time. The recovery rate γ=1.0 indicates the proportion of infected people recovering and turning into a recovery state in a unit of time.

#### 2.2.2. Model Propagation Behavior Characteristics

Based on the dynamic equations above, the SIR model exhibits the following characteristic kinetic behavior: First, the susceptible population declines rapidly due to ongoing infections. Subsequently, the number of infected individuals rises to a peak and then decreases. Finally, after sufficient time has elapsed, both the susceptible and infected populations approach zero, while all individuals transition to the recovered state, marking the end of the transmission process.

In the work, the SIR model serves as the mechanism to generate temporal data from a single evidence source. By simulating the SIR propagation process, we obtain a time series {X(t)} representing the evolution of node states, where $X\left(t\right)={\left[x_{1}\left(t\right),x_{2}\left(t\right),\ldots ,x_{N}\left(t\right)\right]}^{T}$, and $x_{i}\left(t\right)\in \left\{S,I,R\right\}$ denotes the state of node *i* at time *t*. These temporal sequences implicitly encode information about the underlying network connections, thereby forming the essential data foundation for inferring the adjacency matrix based on inter-node state transitions and ultimately reconstructing the complete network topology.

### 2.3. D-S Evidence Theory

D-S evidence theory, also known as the theory of belief functions, was pioneered by Dempster in 1967 and later systematically developed by Shafer in 1976. This theory represents a generalization of classical probability theory, whose core innovation lies in extending the basic event space to its power set and defining the basic probability assignment (BPA) function on this power set [37]. A crucial aspect of applying DST is the quantification of differences or conflicts between evidence, which is often achieved by measuring the distance between BPAs. Recent advancements, such as the method incorporating a penalty coefficient to account for the intrinsic differences between hypotheses themselves, have improved the effectiveness of such distance measures [38]. Beyond measuring conflicts between evidence, a fundamental theoretical question is quantifying the total epistemic uncertainty inherent in the evidence itself and its propagation through fusion processes. For the DCR-based fusion systems central to our framework, rigorous upper bounds on this uncertainty have been established using plausibility entropy, ensuring that the uncertainty within our model is both measurable and bounded [39]. When BPAs are assigned only to singleton subsets, D-S evidence theory reduces to classical probability theory, and Dempster’s combination rule simplifies to Bayes’ rule. This inclusive characteristic endows D-S evidence theory with greater flexibility in representing and handling uncertainty and ignorance, thereby providing a more rigorous and powerful mathematical framework for uncertainty modeling and multi-source information fusion in the network reconstruction task of this paper.

#### 2.3.1. Fundamental Concepts

##### Definition 2.4 (Frame of Discernment)

In evidence theory, let $\Theta =\{\theta_{1},\theta_{2},\ldots ,\theta_{N}\}$ be a finite, complete set of *N* mutually exclusive elements. The power set $2^{\Theta }$, comprising $2^{N}$ subsets, is denoted as

(2) $$\begin{array}{c} 2^{\Theta }=\left\{\emptyset ,\theta_{1},\theta_{2},\ldots ,\theta_{N},\theta_{1}\cup \theta_{2},\ldots ,\theta_{1}\cup \theta_{2}\cup \theta_{3},\ldots ,\Theta \right\} \end{array}$$

Intuitively, the frame of discernment represents the complete set of all possible mutually exclusive outcomes in the problem under study, forming the fundamental universe for evidential reasoning. Within this framework, each subset corresponds to a proposition, reflecting different assertions about the possible states of the problem. By mapping logical propositions and their relationships into set-theoretic objects, the frame of discernment transforms abstract logical concepts into concrete set-based representations. This allows logical operations to be replaced by set operations such as intersection, union, and complement, thereby establishing a rigorous mathematical foundation for reasoning under uncertainty. To address the specific problem of determining whether an edge exists between any two nodes in a complex network, this paper introduces a single-edge frame of discernment Θ={{T},{F},{T,F}}, where *T* represents evidence supporting the presence of an edge, *F* evidence against its existence, and {T,F} denotes the indeterminate state. Its power set $2^{\Theta }=\left\{\emptyset ,\left\{T\right\},\left\{F\right\},\left\{T,F\right\}\right\}$ comprises all possible composite propositions. This frame of discernment reframes the edge inference problem as a process of assigning degrees of belief to these three mutually exclusive propositions, thereby establishing a formal basis for subsequent evidence-based information fusion.

##### Definition 2.5 (Basic Probability Assignment)

For the identification framework Θ, let $2^{\Theta }$ be the power set of Θ. A function $m:2^{\Theta }\to \left[0,1\right]$ is called a *Basic Probability Assignment* if it satisfies:

(3) $$\begin{array}{c} m(\emptyset )=0 \end{array}$$

and

(4) $$\begin{array}{c} \sum_{A\subseteq \Theta }m\left(A\right)=1 \end{array}$$

The value m(A) refers to the degree of support for the proposition *A* under the current evidence. In the study, its core components and their physical meanings are: m({T}): The degree of belief directly supporting the existence of an edge. m({F}): The degree of belief directly supporting the absence of an edge. m(Θ)=m({T,F}): The uncertain belief, representing the portion of belief that cannot be allocated to either *T* or *F* due to incomplete information, explicitly quantifying uncertainty.

##### Definition 2.6 (Belief Function)

Let *m* be a basic probability assignment on Θ. If the function $Bel:2^{\Theta }\to \left[0,1\right]$ satisfies:

(5) $$\begin{array}{c} Bel\left(A\right)=\sum_{B\subseteq A}m\left(B\right),\;A\in 2^{\Theta } \end{array}$$

then Bel(A) is called the belief measure of proposition *A* being true. For singleton propositions *A*, Bel(A)=m(A). The belief function satisfies:

(6) $$\begin{array}{c} Bel(\emptyset )=0,\;Bel(\Theta )=1 \end{array}$$

The belief function represents the degree of trust in proposition *A* being true.

##### Definition 2.7 (Plausibility Function)

Let *m* be a basic probability assignment on Θ. If the function $Pl:2^{\Theta }\to \left[0,1\right]$ satisfies for all $A\in 2^{\Theta }$:

(7) $$\begin{array}{c} Pl\left(A\right)=1-Bel\left(\bar{A}\right)=\sum_{B\cap A\neq \emptyset }m\left(B\right) \end{array}$$

then Pl is called the plausible measure on Θ, and Pl(A) represents the degree of not opposing proposition *A*.

The interpretation of the plausibility function is: Bel(A) represents the degree of trust in *A* being true, $Bel(\bar{A})$ represents the degree of trust in *A* beingfalse, and, therefore, Pl(A) represents the degree of trust in *A* not being false.

From the definitions of basic probability assignment, belief function, and plausibility function, the following relationship holds:

(8) $$\begin{array}{c} Bel(A)\leq m(A)\leq Pl(A),\;\forall A\subseteq \Theta \end{array}$$

That is, Bel(A) and Pl(A) represent the lower and upper bounds of the support degree for proposition *A*, respectively. The interval [Bel(A),Pl(A)] completely describes the belief range for proposition *A*, and its width Pl(A)−Bel(A) intuitively reflects the degree of uncertainty associated with that proposition.

##### Definition 2.8 (Dempster’s Combination Rule)

Dempster’s combination rule is the core mechanism for fusing multiple independent bodies of evidence. For two BPAs $m_{1}$ and $m_{2}$ defined on the same frame Θ, the fused BPA $m=m_{1}⊕m_{2}$ is given by [40]:

(9) $$\begin{array}{c} m\left(A\right)=\frac{1}{1-K}\sum_{B\cap C=A}m_{1}\left(B\right)m_{2}\left(C\right),\;\mathrm{for}\;A\neq \emptyset \end{array}$$

where the conflict coefficient *K* is:

(10) $$\begin{array}{c} K=\sum_{B\cap C=\emptyset }m_{1}\left(B\right)m_{2}\left(C\right) \end{array}$$

The normalization factor 1/(1−K) is applied to exclude the influence of conflicting information between evidence bodies, ensuring that the fusion result remains a valid basic probability assignment (BPA). When K=0, the evidence is completely consistent; when K=1, the evidence is in total conflict and cannot be combined.

#### 2.3.2. Application in Network Reconstruction

This paper innovatively applies D-S evidence theory to the problem of network reconstruction, with its core mechanism operating at two distinct levels:

1. Single-Source Uncertainty Modeling: The time-series data generated from multiple SIR simulations of a single seed node are converted into Basic Probability Assignments (BPAs) according to the edge-existence criterion defined in this work. This process transforms initial, noisy correlation estimates into quantified belief distributions over the propositions T (edge exists), F (edge does not exist), and T,F (uncertain), thereby explicitly modeling the uncertainty inherent in observations from a single information source.

2. Multi-Source Evidence Fusion: To overcome the limited perspective of any single observation point, the BPAs derived from different seed nodes—each having undergone intra-node fusion—are treated as independent bodies of evidence. By applying Dempster’s combination rule to sequentially fuse these evidence sets, the framework effectively integrates complementary structural information from different network regions while concurrently reducing the random uncertainty associated with each individual source. The result is a robust, global belief estimate for the existence of each potential edge, which directly and reliably supports the inference of the network adjacency matrix.

## 3. Network Reconstruction Based on Evidence Theory from Multi-Source Temporal Data

### 3.1. Overview

**Figure 2:**
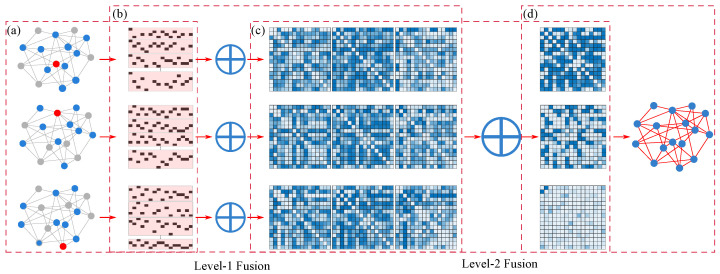
The double-level belief convergence framework process of network reconstruction is as follows: (**a**) Data generation: each single-source information is used as seed nodes for SIR propagation simulation to generate a state timing matrix between nodes; (**b**) Intra-Node Fusion: For each seed node, multiple SIR propagation results are superimposed and integrated into a counting matrix, and the association matrix is obtained after normalization. Finally, the basic probability distribution matrix is generated through D-S evidence theory fusion (support BPA_T_, opposition BPA_F_, uncertainty BPA_TF_). (**c**) Inter-Node Fusion: Use the Dempster synthesis rule to sequentially fuse the BPA matrix obtained corresponding to all single information sources, and finally obtain the adjacent matrix of the reconstruction result through threshold decision-making of BPA_T_. (**d**) Final Decision: The adjacency matrix of the reconstructed network is obtained by applying a threshold to the final BPA_T_ matrix.

As mentioned in Section 2, existing network reconstruction methods exhibit significant limitations in handling local observations, data sparsity, and uncertainty processing. To address these challenges, this chapter details a novel reconstruction method based on the Two-level Belief Fusion Framework (TBFF). This framework infers inter-node connectivity through hierarchical fusion of multi-source temporal data. Figure 2 illustrates the overall workflow:

Intra-Node Fusion: For each selected seed node, an association matrix is generated from multiple independent SIR propagations. The key innovation lies in treating the symmetric parts of this matrix as two independent bodies of evidence, which are then fused using D-S evidence theory to produce a Basic Probability Assignment (BPA) that captures the local perspective of that node.

Inter-Node Fusion: The BPAs derived from different seed nodes are treated as multiple independent evidence sources for second-level fusion. This stage integrates complementary structural information from different regions of the network, ultimately yielding a global BPA regarding the existence of each potential edge.

The two-level fusion structure progressively refines and consolidates evidence, significantly enhancing the robustness of reconstruction under local observation. The following sections elaborate on the detailed implementation of the TBFF.

### 3.2. Data Generation and Association Matrix Construction

This section details the process of generating the foundational data for network reconstruction through SIR propagation simulations from known single-source nodes. The core workflow involves performing multiple independent SIR propagations for each seed node, integrating the resulting temporal data into a count matrix, and subsequently normalizing it to form an association matrix. This matrix serves as the basis for the subsequent intra-node evidence fusion stage.

#### 3.2.1. SIR Propagation Simulation and Time-Series Data Generation

**Figure 3:**
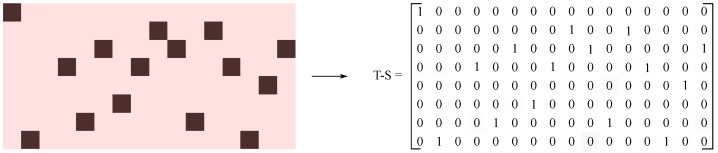
Generation of a Time–State binary matrix based on SIR propagation. The state transfer between nodes in the process of SIR propagation (Figure 1) contains rich information. This information is first expressed as the SIR infection sequence block diagram on the left side of the figure, in which the black square indicates that the node is infected at the corresponding time. In order to facilitate the unified processing of the information obtained from multiple SIR simulations, we store the information obtained in a single matrix form, that is, the time–state binary matrix shown on the right side of the figure. The T-S matrix is formally defined as follows: T-S(i,j)=1 if node *i* is infected at time *j*, and T-S(i,j)=0 if it is susceptible.

Although the SIR model was originally designed to simulate disease transmission, the phenomenon of state transfer between individuals in the process of disease transmission can be extended to the field of network science, which can be specifically used to infer potential connections between network nodes from the dynamics of transmission. This study uses a benchmark network containing 16 nodes and 39 edges to preliminarily verify the method in this article. At the initial moment t=1, node 1 was selected as the seed node for an SIR propagation, and this infection set the infection probability β=0.5 and the recovery rate γ=1.0. When the simulation continues to t=8, the propagation process will naturally end. The results obtained from each propagation simulation are converted into a time–state binary matrix $T\_S\in {\left\{0,1\right\}}^{T\times N}$, so as to facilitate the storage of the information obtained from this infection. See Figure 3, whose formal definition is as follows:

- Temporal dimension: Rows correspond to each moment of the discrete time step t=1,2,…,T
- Spatial dimension: Columns represent network nodes j=1,2,…,N
- State representation: Each element T_S(t,j) indicates the epidemiological state:
  (11) $$\begin{array}{c} T\_S\left(t,j\right)=\left\{\begin{array}{cc} 1, & \mathrm{node}\;j\;\mathrm{is}\;\mathrm{infected}\;\mathrm{at}\;\mathrm{time}\;t\\ 0, & \mathrm{node}\;j\;\mathrm{is}\;\mathrm{susceptible}/\mathrm{recovered}\;\mathrm{at}\;\mathrm{time}\;t \end{array}\right. \end{array}$$

#### 3.2.2. Basic Criterion for Edge Existence and Count Matrix Construction

**Figure 4:**
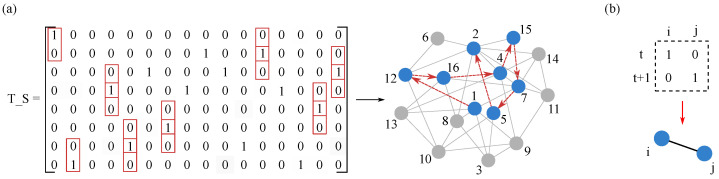
Inferring potential network edges from the Time–State (T-S) matrix. (**a**) The T-S matrix from Figure 3. A hypothetical infection path from node 1 to node 2 over 8 time steps is highlighted, illustrating how the sequence of infections can suggest a direct connection. (**b**) The proposed criterion for edge inference: an observed state transition where node *i* is infected at time *t* and node *j* becomes infected at time t+1 provides supporting evidence for a potential edge between *i* and *j*. This criterion can reveal the real connection in the network. However, the information obtained by a single SIR simulation still contains a lot of uncertainty and conflicting information, as shown in the unparsed state in (**a**).

Based on the dynamic characteristics of SIR propagation, we put forward the following criteria for judging whether there is a connection between nodes: as shown in Figure 4b, if node *i* is in an infected state at the moment *t* and node *j* is in a healthy state, and at the moment t+1, node *i* returns to a healthy state and node *j* turns into an infected state. Such a state transfer is considered to have a connection between the supporting nodes *i* and *j*. Through this guideline, the timing information directly transmitted from node *i* to node *j* during the infection process can be obtained. As shown in Figure 4a, a potential propagation path from node 1 to node 5 is extracted from the T-S matrix through this criterion, which visually shows that the simulation of SIR propagation on the network has the ability to reveal the network topology.

In order to facilitate the quantification of this dynamic information as evidence later, we will conduct *M* independent SIR simulations for each selected seed node. Events satisfying the above criterion in each resulting T-S matrix are counted, and the counts from the *M* matrices are summed to construct a Count Matrix ($M_{C}$). The element $M_{C}\left(i,j\right)$ represents the total number of times infection is observed to propagate directly from node *i* to node *j* across the *M* independent trials.

#### 3.2.3. Association Matrix Calculation

To eliminate the dimensional influence of absolute counts and standardize the data scale to the interval [0,1], the count matrix $M_{C}$ is normalized to produce the Association Matrix ($M_{R}$). The calculation formula is:

(12) $$\begin{array}{c} M_{R}\left(i,j\right)=\frac{M_{C}\left(i,j\right)}{\max(M_{C})} \end{array}$$

where $\max(M_{C})$ is the maximum value among all elements in the count matrix $M_{C}$. After normalization, the value of $M_{R}\left(i,j\right)\in \left[0,1\right]$ intuitively reflects the relative support strength for the existence of a directed edge from node *i* to *j*.

**Figure A1:**
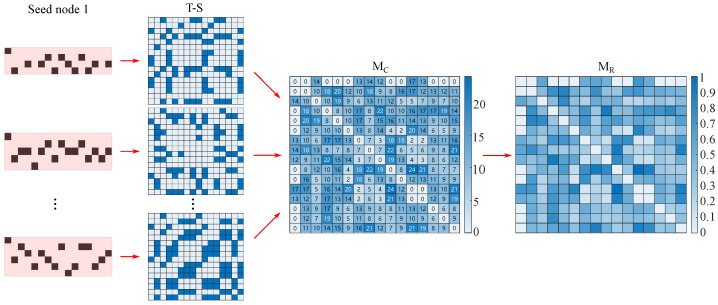
(Appendix) Generation of the association matrix $M_{R}$ from SIR simulations. This figure details the process outlined in Section 3.1, showing the aggregation of time–state matrices into a count matrix $M_{C}$ and its subsequent normalization to $M_{R}$.

For a concrete, step-by-step visual example of generating $M_{R}$ from SIR simulations, please refer to Figure A1 in the Appendix A. At this stage, the information obtained by each seed node through *M* simulations is integrated into the association matrix $M_{R}$ through the above process. This matrix serves as the direct input for the subsequent Level-1 evidence fusion.

### 3.3. First-Level Fusion: Generating Intra-Node Evidence Through Symmetric Fusion

**Figure A2:**
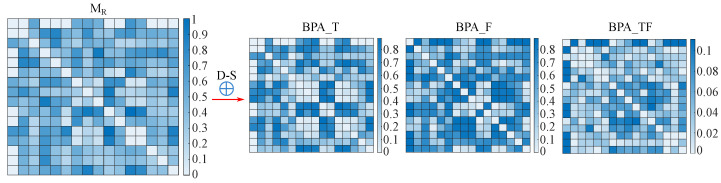
(Appendix) Intra-node fusion: from association matrix to BPA matrices. This figure illustrates the first-level fusion process described in Section 3.2, demonstrating the conversion of asymmetric correlation information into symmetric Basic Probability Assignments.

This section aims to transform the correlation matrix $M_{R}$ corresponding to a single seed node into a set of Basic Probability Assignments (BPAs). In undirected network reconstruction, the correlation information of node pairs (i,j) and (j,i) should collaboratively characterize the same potential edge. However, in practical observations, evidence from these two directions may exhibit uncertainty and conflict due to the stochasticity of propagation. To fully leverage this symmetry and enhance evidence quality, we innovatively treat the upper triangular and lower triangular parts of the correlation matrix as two independent evidence sources and fuse them using D-S evidence theory. A detailed visual workflow of this intra-node fusion process, using node 1 of the benchmark network as an example, is provided in Figure A2 of the Appendix A.

#### 3.3.1. Evidence Source Definition and Initial Belief Calculation

The evidence of in-node fusion comes from the asymmetric node association information in the association matrix $M_{R}$. For each pair of directed nodes (i,j) (where i≠j), we define the information in the upper and lower triangular parts of the associated matrix as two sources of evidence that capture bidirectional propagation information:

- Evidence source $E_{ij}$: The impact of node *i* on node *j* during the propagation process is assigned by the associated element $M_{R}\left(i,j\right)$;
- Evidence source $E_{ji}$: The influence of node *j* on node *i* during propagation is assigned by $M_{R}\left(j,i\right)$.

The two-way evidence source definition emphasizes that even in the topology reconstruction of undirected networks, the two-way information between nodes should be considered to improve and optimize the reconstruction results. For each pair $\left(E_{ij},E_{ji}\right)$, we calculate their initial belief assignments for the proposition ‘edge existence’ using their correlation strength values as input. The calculation is based on the deviation of these values from the global maximum $R_{\max}$, minimum $R_{\min}$, and median $R_{\mathrm{mid}}=\left(R_{\max}+R_{\min}\right)/2$ of the matrix. This conversion method, which quantifies uncertainty through deviation from global statistics, draws on the idea established by [41] for transforming local network association strength into evidence bodies. Its effectiveness in handling uncertainty within complex network structures has been previously validated. Specifically, taking evidence source $E_{ij}$ as an example, its initial belief is calculated as follows:

(13) $$\begin{array}{cc} {\mathrm{Summ}}_{ij} & =\;|M_{R}\left(i,j\right)-R_{\max}\left|+\right|M_{R}\left(i,j\right)-R_{\min}\left|+\right|M_{R}\left(i,j\right)-R_{\mathrm{mid}}| \end{array}$$

(14) $$\begin{array}{cc} T_{ij} & =\;|M_{R}\left(i,j\right)-R_{\min}|/{\mathrm{Summ}}_{ij} \end{array}$$

(15) $$\begin{array}{cc} F_{ij} & =\;|M_{R}\left(i,j\right)-R_{\max}|/{\mathrm{Summ}}_{ij} \end{array}$$

(16) $$\begin{array}{cc} TF_{ij} & =\;|M_{R}\left(i,j\right)-R_{\mathrm{mid}}|/{\mathrm{Summ}}_{ij} \end{array}$$

Here, *T*, *F*, and TF represent the initial belief for supporting, opposing, and being uncertain about edge existence, respectively. Similarly, for evidence source $E_{ji}$, we compute $T_{ji}$, $F_{ji}$, and $TF_{ji}$.

#### 3.3.2. Dempster Evidence Fusion and BPA Generation

The two sets of initial beliefs $\left(T_{ij},F_{ij},TF_{ij}\right)$ and $\left(T_{ji},F_{ji},TF_{ji}\right)$ are treated as two independent bodies of evidence concerning the same proposition (an edge exists between nodes *i* and *j*) and fused using Dempster’s combination rule. First, the conflict coefficient *K* between these two bodies of evidence is calculated:

(17) $$K=T_{ij}\cdot F_{ji}+T_{ji}\cdot F_{ij}$$

Subsequently, normalized fusion is performed to obtain the final, combined BPA values for the node pair (i,j):

(18) $$\begin{array}{cc} {\mathrm{BPA}}_{T}\left(i,j\right) & =T_{ij}T_{ji}+T_{ij}TF_{ji}+TF_{ij}T_{ji}/\left(1-K\right) \end{array}$$

(19) $$\begin{array}{cc} {\mathrm{BPA}}_{F}\left(i,j\right) & =F_{ij}F_{ji}+F_{ij}TF_{ji}+TF_{ij}F_{ji}/\left(1-K\right) \end{array}$$

(20) $$\begin{array}{cc} {\mathrm{BPA}}_{TF}\left(i,j\right) & =TF_{ij}TF_{ji}/\left(1-K\right) \end{array}$$

Since we are reconstructing an undirected network, we set BPA(j,i)=BPA(i,j), meaning the BPA matrix is symmetric. Through the above operations on all asymmetric node pairs in the correlation matrix $M_{R}$, we obtain a complete set of BPAs representing the local perspective of the seed node, namely the support matrix ${\mathrm{BPA}}_{T}$, opposition matrix ${\mathrm{BPA}}_{F}$, and uncertainty matrix ${\mathrm{BPA}}_{TF}$. This output will serve as input for the second-level fusion.

### 3.4. Second-Level Fusion: Inter-Node Fusion and Decision

After completing the first-level fusion, we obtain BPA sets corresponding to *S* seed nodes, where each set contains $\left\{{\mathrm{BPA}}_{T}^{\left(s\right)},{\mathrm{BPA}}_{F}^{\left(s\right)},{\mathrm{BPA}}_{TF}^{\left(s\right)}\right\}$ for s=1,2,…,S. This section aims to integrate this evidence from different local perspectives of the network through second-level fusion to derive globally consistent belief estimates and ultimately reconstruct the network adjacency matrix.

#### 3.4.1. Evidence Synthesis Among Nodes Based on Sequential Fusion

We carry out orthogonal fusion of evidence from *S* seed nodes sequentially. The BPA from the first seed node serves as the initial global BPA. Subsequent seed nodes are then fused iteratively using Dempster’s rule. The BPA from the first seed node initializes the global BPA:

(21) $$\begin{array}{cc} {\mathrm{BPA}}_{T}^{G(1)} & =\;{\mathrm{BPA}}_{T}^{\left(1\right)}, \end{array}$$

(22) $$\begin{array}{cc} {\mathrm{BPA}}_{F}^{G(1)} & =\;{\mathrm{BPA}}_{F}^{\left(1\right)}, \end{array}$$

(23) $$\begin{array}{cc} {\mathrm{BPA}}_{TF}^{G(1)} & =\;{\mathrm{BPA}}_{TF}^{\left(1\right)}. \end{array}$$

For each subsequent seed node s=2,3,…,S, the BPA $\left\{{\mathrm{BPA}}_{T}^{\left(s\right)},{\mathrm{BPA}}_{F}^{\left(s\right)},{\mathrm{BPA}}_{TF}^{\left(s\right)}\right\}$ is fused with the current global BPA $\left\{{\mathrm{BPA}}_{T}^{G(s-1)},{\mathrm{BPA}}_{F}^{G(s-1)},{\mathrm{BPA}}_{TF}^{G(s-1)}\right\}$ for each node pair (i,j). The conflict coefficient $K_{s}\left(i,j\right)$ is calculated as:

(24) $$K_{s}\left(i,j\right)={\mathrm{BPA}}_{T}^{G(s-1)}\left(i,j\right)\cdot {\mathrm{BPA}}_{F}^{\left(s\right)}\left(i,j\right)+{\mathrm{BPA}}_{F}^{G(s-1)}\left(i,j\right)\cdot {\mathrm{BPA}}_{T}^{\left(s\right)}\left(i,j\right).$$

Then, the updated global BPA components are obtained via Dempster’s rule:

(25) $$\begin{array}{cc} M & ={\mathrm{BPA}}_{T}^{G(s-1)}\left(i,j\right)\left[{\mathrm{BPA}}_{T}^{\left(s\right)}\left(i,j\right)+{\mathrm{BPA}}_{TF}^{\left(s\right)}\left(i,j\right)\right]+{\mathrm{BPA}}_{TF}^{G(s-1)}\left(i,j\right){\mathrm{BPA}}_{T}^{\left(s\right)}\left(i,j\right), \end{array}$$

(26) $$\begin{array}{cc} N & ={\mathrm{BPA}}_{F}^{G(s-1)}\left(i,j\right)\left[{\mathrm{BPA}}_{F}^{\left(s\right)}\left(i,j\right)+{\mathrm{BPA}}_{TF}^{\left(s\right)}\left(i,j\right)\right]+{\mathrm{BPA}}_{TF}^{G(s-1)}\left(i,j\right){\mathrm{BPA}}_{F}^{\left(s\right)}\left(i,j\right), \end{array}$$

(27) $$\begin{array}{cc} {\mathrm{BPA}}_{T}^{G(s)}\left(i,j\right) & =\frac{M}{1-K_{s}\left(i,j\right)}, \end{array}$$

(28) $$\begin{array}{cc} {\mathrm{BPA}}_{F}^{G(s)}\left(i,j\right) & =\frac{N}{1-K_{s}\left(i,j\right)}, \end{array}$$

(29) $$\begin{array}{cc} {\mathrm{BPA}}_{TF}^{G(s)}\left(i,j\right) & =\frac{{\mathrm{BPA}}_{TF}^{G(s-1)}\left(i,j\right){\mathrm{BPA}}_{TF}^{\left(s\right)}\left(i,j\right)}{1-K_{s}\left(i,j\right)}. \end{array}$$

After processing all *S* seed nodes, the final global BPA is:

(30) $$\begin{array}{cc} {\mathrm{BPA}}_{T}^{\mathrm{final}} & =\;{\mathrm{BPA}}_{T}^{G(S)}, \end{array}$$

(31) $$\begin{array}{cc} {\mathrm{BPA}}_{F}^{\mathrm{final}} & =\;{\mathrm{BPA}}_{F}^{G(S)}, \end{array}$$

(32) $$\begin{array}{cc} {\mathrm{BPA}}_{TF}^{\mathrm{final}} & =\;{\mathrm{BPA}}_{TF}^{G(S)}. \end{array}$$

**Figure A3:**
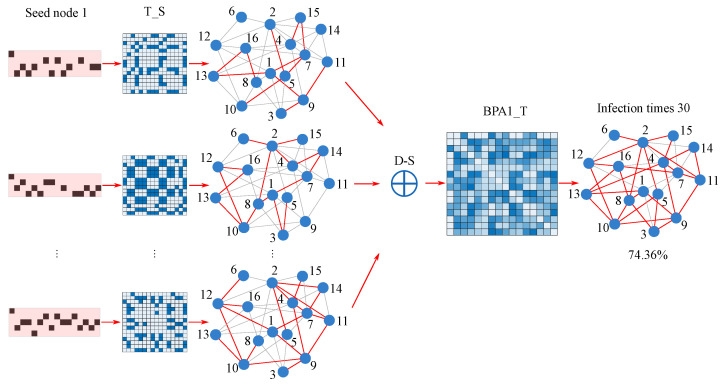
(Appendix) Inter-node fusion and final decision. This figure demonstrates the second-level sequential fusion of BPA sets from multiple seed nodes and the final threshold-based reconstruction of the adjacency matrix, as detailed in Section 3.3.

This sequential fusion approach ensures clarity and scalability in the evidence synthesis process. The complete workflow of the second-level fusion, using nodes 1, 2, and 3 of the 16-node network as examples, is illustrated in Figure A3 in the Appendix A. It demonstrates the sequential fusion of BPA matrices and the final reconstruction of the adjacency matrix.

#### 3.4.2. Final Decision and Adjacency Matrix Generation

Based on the global BPA obtained after fusion, we set a belief threshold θ (e.g., θ=0.7) on ${\mathrm{BPA}}_{T}$ to determine the adjacency matrix *A* of the reconstructed network. For each node pair (i,j) where i≠j, the decision rule is:

(33) $$A\left(i,j\right)=\left\{\begin{array}{cc} 1, & \mathrm{if}\;{\mathrm{BPA}}_{T}^{\mathrm{final}}\left(i,j\right)>\theta \\ 0, & \mathrm{otherwise} \end{array}\right.$$

**Figure 5:**
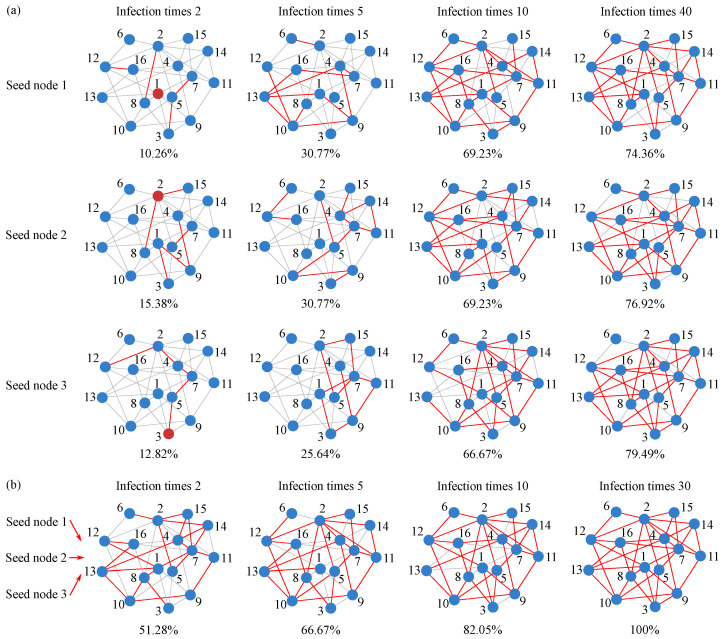
Comparative analysis of network topology reconstruction under intra-node and inter-node fusion. (**a**) Reconstruction results from intra-node fusion. Each row displays the topology independently reconstructed from seed node 1, 2, or 3, respectively. Each column corresponds to a different number of SIR simulations: M=2,5,10,40. By comparing the 12 sub-figures horizontally and vertically, we observe that although reconstruction accuracy generally improves with more simulations, the improvement plateaus at M=40. This indicates the inherent limitation of reconstructing a complete network topology from a single observation perspective. (**b**) Reconstruction results from inter-node fusion. The BPAs derived from nodes 1, 2, and 3 at M=2,5,10,30 are sequentially fused using orthogonal combination. The resulting topology not only surpasses the accuracy achievable from any single seed node but also recovers key connections that are invisible from any individual observation perspective.

Since the BPA matrices are symmetric, the resulting adjacency matrix *A* is also symmetric, which aligns with the undirected nature of the network being reconstructed. The complete workflow from evidence fusion to final network reconstruction is visually summarized in Figure 5, illustrating the transformation of multiple local evidence into a globally consistent network structure.

### 3.5. Algorithm Summary and Complexity Analysis

To clearly present the proposed two-level belief fusion method, this section first summarizes the complete workflow in pseudocode form, followed by a computational complexity analysis to explore its potential for large-scale network applications.

#### 3.5.1. Algorithm Pseudocode

Algorithm 1 outlines the complete process from data generation to final decision, incorporating the first-level fusion (intra-node symmetric fusion) from Section 3.3 and the second-level fusion (inter-node sequential fusion) from Section 3.4.
**Algorithm 1** Two-Level Belief Fusion for Network Reconstruction**Require:**   ▹ Network node set *V* (|V|=N)   ▹ Seed node set $S=\{s_{1},s_{2},\ldots ,s_{S}\}$   ▹ SIR parameters: β, γ, *T*, *M*   ▹ Decision threshold θ **Ensure:**   ▹ Reconstructed adjacency matrix A (N×N)
  1:**Phase 1: Data Generation & Intra-Node Fusion**  2:**for each** seed node s∈S **do**  3:    $M_{C}^{s}\leftarrow 0^{N\times N}$  4:    **for** m=1 **to** *M* **do**  5:        TS←SIR_Simulation(s,β,γ,T)  6:        Update $M_{C}^{s}$ with state transitions in TS  7:    **end for**  8:    $M_{R}^{s}\leftarrow M_{C}^{s}/\max\left(M_{C}^{s}\right)$                       ▹ Normalize  9:    ${\mathrm{BPA}}_{T}^{s},{\mathrm{BPA}}_{F}^{s},{\mathrm{BPA}}_{TF}^{s}\leftarrow 0^{N\times N}$10:    **for each** (i,j) where i<j **do**11:        $\left(T_{ij},F_{ij},TF_{ij}\right)\leftarrow \mathrm{ComputeBelief}\left(M_{R}^{s}\left(i,j\right)\right)$12:        $\left(T_{ji},F_{ji},TF_{ji}\right)\leftarrow \mathrm{ComputeBelief}\left(M_{R}^{s}\left(j,i\right)\right)$13:        $K\leftarrow T_{ij}F_{ji}+T_{ji}F_{ij}$                         ▹ Conflict14:        ${\mathrm{BPA}}_{T}^{s}\left(i,j\right)\leftarrow \left(T_{ij}T_{ji}+T_{ij}TF_{ji}+TF_{ij}T_{ji}\right)/\left(1-K\right)$15:        ${\mathrm{BPA}}_{F}^{s}\left(i,j\right)\leftarrow \left(F_{ij}F_{ji}+F_{ij}TF_{ji}+TF_{ij}F_{ji}\right)/\left(1-K\right)$16:        ${\mathrm{BPA}}_{TF}^{s}\left(i,j\right)\leftarrow \left(TF_{ij}TF_{ji}\right)/\left(1-K\right)$17:        ${\mathrm{BPA}}_{T}^{s}\left(j,i\right),{\mathrm{BPA}}_{F}^{s}\left(j,i\right),{\mathrm{BPA}}_{TF}^{s}\left(j,i\right)\leftarrow {\mathrm{BPA}}_{T}^{s}\left(i,j\right),{\mathrm{BPA}}_{F}^{s}\left(i,j\right),{\mathrm{BPA}}_{TF}^{s}\left(i,j\right)$18:    **end for**19:**end for**20:**Phase 2: Inter-Node Fusion & Decision**21:${\mathrm{BPA}}_{T}^{G},{\mathrm{BPA}}_{F}^{G},{\mathrm{BPA}}_{TF}^{G}\leftarrow {\mathrm{BPA}}_{T}^{s_{1}},{\mathrm{BPA}}_{F}^{s_{1}},{\mathrm{BPA}}_{TF}^{s_{1}}$22:**for** k=2 **to** *S* **do**23:    **for each** (i,j) where i≠j **do**24:        $K\leftarrow {\mathrm{BPA}}_{T}^{G}\left(i,j\right)\cdot {\mathrm{BPA}}_{F}^{s_{k}}\left(i,j\right)+{\mathrm{BPA}}_{F}^{G}\left(i,j\right)\cdot {\mathrm{BPA}}_{T}^{s_{k}}\left(i,j\right)$25:        ${\mathrm{BPA}}_{T}^{G}\left(i,j\right)\leftarrow \frac{{\mathrm{BPA}}_{T}^{G}\left(i,j\right){\mathrm{BPA}}_{T}^{s_{k}}\left(i,j\right)+{\mathrm{BPA}}_{T}^{G}\left(i,j\right){\mathrm{BPA}}_{TF}^{s_{k}}\left(i,j\right)+{\mathrm{BPA}}_{TF}^{G}\left(i,j\right){\mathrm{BPA}}_{T}^{s_{k}}\left(i,j\right)}{1-K}$26:        ${\mathrm{BPA}}_{F}^{G}\left(i,j\right)\leftarrow \frac{{\mathrm{BPA}}_{F}^{G}\left(i,j\right){\mathrm{BPA}}_{F}^{s_{k}}\left(i,j\right)+{\mathrm{BPA}}_{F}^{G}\left(i,j\right){\mathrm{BPA}}_{TF}^{s_{k}}\left(i,j\right)+{\mathrm{BPA}}_{TF}^{G}\left(i,j\right){\mathrm{BPA}}_{F}^{s_{k}}\left(i,j\right)}{1-K}$27:        ${\mathrm{BPA}}_{TF}^{G}\left(i,j\right)\leftarrow \frac{{\mathrm{BPA}}_{TF}^{G}\left(i,j\right)\cdot {\mathrm{BPA}}_{TF}^{s_{k}}\left(i,j\right)}{1-K}$28:    **end for**29:**end for**30:**Final Decision**31:$A\leftarrow 0^{N\times N}$32:**for each** (i,j) where i≠j **do**33:    **if** ${\mathrm{BPA}}_{T}^{G}\left(i,j\right)>\theta$ **then**34:        A(i,j)←135:    **end if**36:**end for**37:**return** A


#### 3.5.2. Complexity Analysis

The content of this section is to analyze the computational complexity of the algorithm in this article. The main parameters are defined as follows: *N* represents the number of network nodes, *S* represents the number of seed nodes, and *M* represents the number of simulations for each seed node. The analysis is performed stage by stage.

- Data Generation Stage: The complexity of a single SIR simulation is related to the network structure. For analysis purposes, we consider the upper bound where the propagation might traverse many potential edges. Thus, the complexity per simulation can be considered as $O(N^{2})$ for a dense network. Since we perform *M* simulations for each of the *S* seed nodes, the total complexity of this stage is $O(S\cdot M\cdot N^{2})$.
- The First-Level Fusion Stage: This stage processes the association matrix for each seed node. The fusion operation needs to be performed for all $O(N^{2})$ node pairs independently. Therefore, the computational cost for this stage is $O(S\cdot N^{2})$.
- The Second-Level Fusion Stage: In this stage, we sequentially fuse the BPA matrices from all *S* seed nodes. Each sequential fusion operation also requires traversing all $O(N^{2})$ node pairs. Consequently, the complexity of this stage is $O(S\cdot N^{2})$.
- Decision-making Phase: The final step involves applying a threshold to the global belief matrix, which has a complexity of $O(N^{2})$.

From the above analysis, the overall time complexity of the proposed method is dominated by the data generation stage, which is $O(S\cdot M\cdot N^{2})$. It is important to note that in our proposed framework, the number of seed nodes *S* and the number of simulations per seed *M* are typically set to small constants (e.g., 3 to 5) to meet the local observation constraint. Therefore, the time complexity effectively scales polynomially with respect to the network size *N*, specifically as $O(N^{2})$. This complexity is comparable to that of calculating a simple correlation matrix and is often more efficient than many model-based inference methods, such as those relying on iterative optimization.

Regarding space complexity, the algorithm primarily needs to store the association matrices and the BPA matrices for the *S* seed nodes during processing. Each of these is an N×N matrix. Therefore, the dominant space complexity is $O(S\cdot N^{2})$. After the final fusion, only the global N×N belief matrix needs to be retained, requiring $O(N^{2})$ space. Given that *S* is a small constant, the memory requirement is manageable for large-scale networks.

In summary, the proposed two-level belief fusion framework exhibits polynomial time and space complexity, confirming its theoretical feasibility for application to large-scale complex network reconstruction problems under local observation conditions.

## 4. Experiments and Results Analysis

This chapter systematically evaluates the network reconstruction performance of the proposed two-level evidence fusion framework. First, in Section 4.1, we conduct controlled experiments on a benchmark network with 16 nodes and 39 edges to investigate the respective contributions of intra-node and inter-node fusion to reconstruction accuracy, as well as the combined effect of both stages on the overall reconstruction outcome. Second, in Section 4.2, we apply the proposed method to several real-world networks and compare its performance with representative existing reconstruction methods, thereby assessing the framework’s applicability and robustness in practical complex systems. The experiments presented in this chapter not only validate the feasibility and accuracy of the algorithm but also empirically demonstrate the methodological insight that multi-source evidence fusion can progressively uncover the true structure of a system.

### 4.1. Feasibility Validation on Benchmark Networks

This section systematically validates the effectiveness of the proposed two-level belief fusion framework using a benchmark network with 16 nodes and known topology. The well-defined structure and moderate scale of this network provide an ideal testbed for achieving two primary objectives: (1) to precisely isolate and quantify the individual contributions of intra-node and inter-node fusion to reconstruction accuracy, and (2) to facilitate complete tracing and visualization of the entire inference chain from sparse local observations to the complete global topology, thereby intuitively revealing the working mechanism of our approach.

#### 4.1.1. Experimental Setup

All experiments in this subsection are conducted on the 16-node benchmark network introduced in Section 3. Figure 5 visually illustrates the reconstruction performance. Figure 5a shows the reconstruction results obtained separately from seed nodes 1, 2, and 3 under different simulation counts M={2,5,10,40}. Each panel corresponds to a specific *M* value, demonstrating how reconstruction quality from a single seed node improves with more simulation data. Figure 5b presents the final reconstructed topology, obtained by orthogonally fusing the BPA matrices derived from the three seed nodes at M={2,5,10,30}. This fusion process integrates evidence from multiple local perspectives to yield a more robust global reconstruction.

To quantitatively evaluate the reconstruction performance, we employ the true positive rate (TPR) and false positive rate (FPR) as metrics. The TPR measures the proportion of actual edges correctly identified, while the FPR indicates the proportion of non-edges mistakenly classified as edges. They are defined as:

(34) $$\begin{array}{cc} \mathrm{TPR} & =\frac{\mathrm{TP}}{\mathrm{TP}+\mathrm{FN}} \end{array}$$

(35) $$\begin{array}{cc} \mathrm{FPR} & =\frac{\mathrm{FP}}{\mathrm{TN}+\mathrm{FP}} \end{array}$$

Here, TP, FN, FP, and TN denote true positives, false negatives, false positives, and true negatives, respectively.

The experimental parameters follow the settings established in Section 3: the infection probability is set to β=0.5, and the recovery rate to γ=1.0. A recovery rate of γ=1.0 ensures that an infected node recovers in the subsequent time step and does not participate in further propagation, thereby simplifying the dynamic analysis. For the final decision, a belief threshold θ=0.7 is applied to the global ${\mathrm{BPA}}_{T}$ matrix. This value is selected to retain edges with strong evidential support while minimizing reconstruction errors in the final adjacency matrix.

#### 4.1.2. Effectiveness Analysis of Intra-Node Fusion

**Figure 6:**
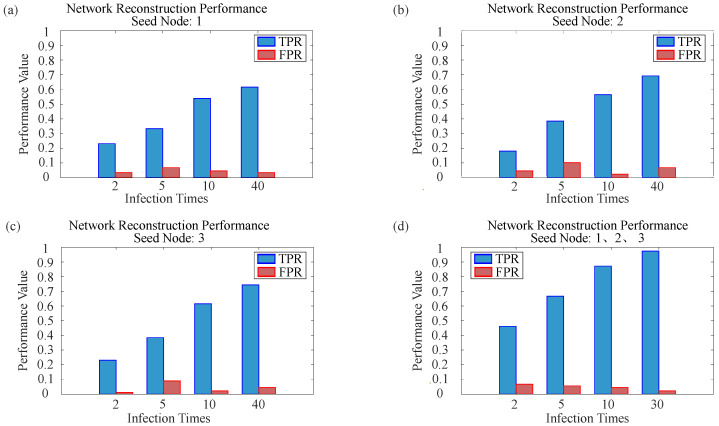
Comparative analysis of reconstruction accuracy under single-node observation versus multi-node fusion. (**a**–**c**) Take node 1, node 2 and node 3 as the only seed nodes, respectively, and perform under different SIR simulations (M=2,5,10,40). With the increase of *M*, the improvement of TPR is limited, especially after M=10, indicating that the improvement of the quality of evidence of the increasing number of SIR simulations under the single-source observation perspective is limited. (**d**) The bar chart of the inter-node fusion performance obtained by orthogonal fusion obtained by reconstructing the three nodes as the results of the seed nodes under M=2,5,10,30. Unlike the in-node fusion results, even under a limited number of SIR infection simulations (M=2), the reconstruction results of inter-node fusion results are better than the reconstruction results obtained in the in-node fusion phase at a higher number of simulations, which proves the need to increase the inter-node fusion steps.

To further validate the effectiveness of intra-node fusion in processing uncertain information, we systematically examine how the number of SIR simulations affects the accuracy of sub-network reconstruction from different observational perspectives. Using nodes 1, 2, and 3 of the 16-node, 39-edge network as seed nodes, we analyze the changes in reconstruction metrics when the number of simulations *M* is set to 2, 5, 10, and 40. A single SIR simulation starting from one seed node often yields highly uncertain information due to the stochastic nature of propagation. The innovation of intra-node fusion lies in mitigating this uncertainty by combining multiple SIR runs with D-S theory, thereby enhancing the reliability of evidence obtained from a single observation perspective. Figure 6a–c presents bar charts of the TPR and FPR of the reconstruction results from the three single-source perspectives (nodes 1, 2, and 3) across different simulation counts *M*.

The figure reveals that, although the three seed nodes represent distinct local observation perspectives and yield different reconstruction accuracies across SIR simulations, their TPRs exhibit highly similar trends as *M* increases. Specifically, TPR rises rapidly at small *M* and then grows more slowly, indicating that increasing the number of simulations effectively enhances evidence quality through intra-node fusion. A closer comparison shows that the largest TPR gain occurs when *M* increases from 2 to 10, demonstrating that even a modest number of additional simulations can substantially improve evidence reliability via intra-node fusion. In contrast, further increasing *M* from 10 to 40 yields only marginal improvements, suggesting that beyond a certain point, adding more simulations has limited impact on evidence quality.

Thus, while intra-node fusion can improve the evidence quality from any single observation perspective, its effectiveness is bounded. This finding highlights a trade-off between computational cost and evidence refinement and offers practical guidance for parameter selection in real-network reconstruction.

#### 4.1.3. Effectiveness Analysis of Inter-Node Fusion

This section examines whether inter-node fusion can integrate limited local observations to yield a more robust global reconstruction that surpasses the performance of any single node, given the diminishing returns observed in intra-node fusion. Building on the results from Section 4.1.2, we take the BPA results from nodes 1, 2, and 3 at M={2,5,10,30} and their corresponding reconstruction metrics shown in Figure 6b. For each *M*, we sequentially fuse the BPAs from all three seed nodes and compare the fused performance with that of each individual node under the same *M*. Figure 6d displays the TPR and FPR bar charts obtained after inter-node fusion under the four simulation counts.

Across all four simulation levels (M=2,5,10,30), the TPR after inter-node fusion is markedly higher than that achieved by intra-node fusion alone. Notably, even with very limited observational data (M=2), inter-node fusion outperforms intra-node fusion with more simulations (M=5). Although both intra-node and inter-node reconstruction improve as *M* increases, the superiority of inter-node fusion remains consistent and substantial.

These results strongly confirm the importance of the inter-node fusion step: it effectively integrates evidence from multiple observational perspectives, produces a more comprehensive and accurate global topology, and fundamentally overcomes the inherent limitations of any single-node viewpoint.

### 4.2. Real-World Network Reconstruction Performance Evaluation

To comprehensively verify that the proposed two-level belief fusion framework (TBFF) remains robust and practical for real-world network reconstruction, this section applies it to five real networks with varying scales and topological characteristics. The five networks are the Karate Club Network, the American Airlines Network, the Copenhagen Twitter Forwarding Network, the German Highway Network, and the Yeast Protein Interaction Network [35,42,43,44,45]. During reconstruction, the SIR propagation parameters are tailored to each network’s structure to ensure the epidemic can sufficiently explore the network and generate informative observational data. Later in this chapter, the reconstruction performance of TBFF will be compared with that of a representative baseline method (NRA) on each real network, thereby validating the framework’s effectiveness in handling uncertain information present in real data.

#### 4.2.1. Case Study: Karate Club Network

**Figure 7:**
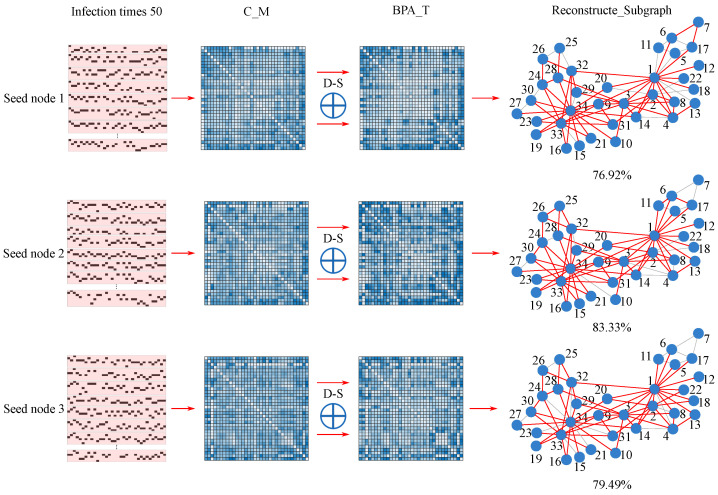
Network topology reconstruction from a single-node observation perspective. This figure shows the reconstruction of the Karate Club network using node 1, node 2, and node 3 as independent single-source observers. For each seed node, reconstruction begins with the temporal data generated by 50 SIR simulations. The connected edges shown in each subgraph are then determined based on the BPA obtained after intra-node fusion. These results reveal the inherent limitations on reconstruction accuracy when only a single observation perspective is available.

**Figure 8:**
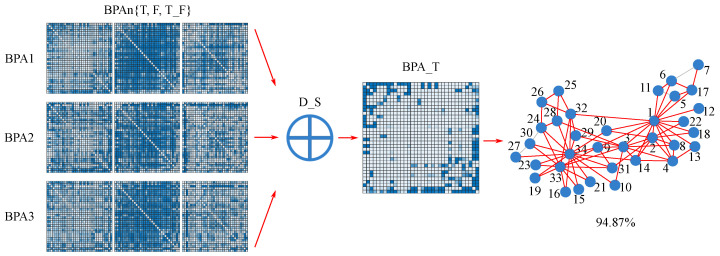
Reconstruction of the Karate Club Network Based on Multi-Source Evidence Fusion. This figure demonstrates the substantial improvement in reconstruction accuracy achieved through inter-node evidence fusion. For the 34-node Karate Club network, nodes 1, 2, and 3 are selected as three independent information sources, each providing temporal data from 50 independent SIR simulations. The Basic Probability Assignments (BPAs) corresponding to these nodes are sequentially fused using Dempster’s combination rule, yielding a final global BPA. Applying the predetermined threshold to the resulting ${\mathrm{BPA}}_{T}$ matrix produces the reconstructed topology shown here. The achieved reconstruction accuracy of 94.87% notably exceeds the accuracy obtained from any single node in Figure 7, reinforcing the necessity of multi-source fusion for obtaining high-precision network reconstruction results.

**Figure 9:**
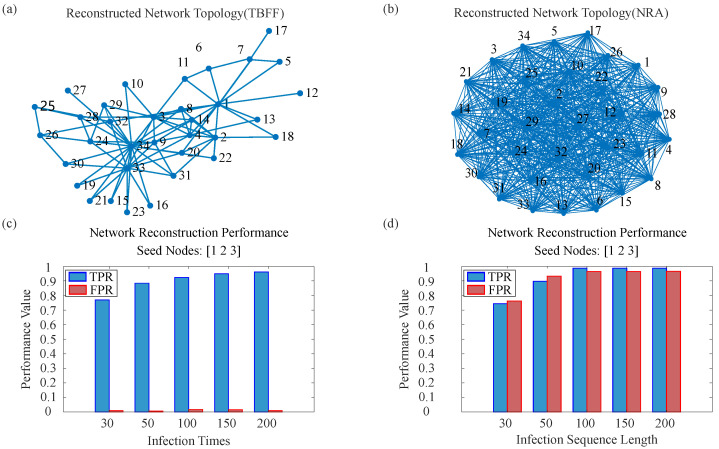
Comparison of reconstruction performance on the Karate Club network. (**a**) The topology reconstructed by the proposed TBFF framework accurately restores the two main community structures and the key bridging connections between them. (**b**) The topology reconstructed by the baseline NRA method blurs community boundaries and contains numerous spurious connections, resulting in a cluttered structure. (**c**) Bar charts of TPR and FPR for three seed nodes under different simulation counts *M*. TPR rises notably with increasing *M*, while FPR remains consistently low, reflecting both high recall and high precision. (**d**) TPR and FPR of the baseline method under different observation sequences. The persistently high FPR—often exceeding the TPR—indicates severe over-fitting and excessive false inference. Subfigures (**b**,**d**) together show that the baseline method fails to recover the network’s essential structure and produces a disordered set of edges. In contrast, the proposed TBFF framework achieves a reconstruction that is both topologically faithful and robust.

In this part, we first examine the reconstruction of the well-known Karate Club network in detail. Given the moderate scale of this network, we set the infection probability to β=0.5 and the decision threshold to θ=0.7 for all SIR simulations. Figure 7 and Figure 8 illustrate the two key evidence fusion stages—intra-node and inter-node fusion—visualizing the complete workflow from raw temporal data to the final reconstructed topology. Finally, Figure 9 directly compares the reconstruction results of our framework with those of an existing baseline method (NRA).

#### 4.2.2. First-Level Fusion: From Random Observations to Local Evidence

At this stage, nodes 1, 2, and 3 serve as seed nodes to initiate SIR propagation simulations, generating the raw temporal-state sequences required for the first-level (intra-node) evidence fusion. Each seed node undergoes 50 independent SIR runs to ensure statistical robustness of the observed infection patterns. The detailed workflow for processing this simulation data—from time-series aggregation to correlation matrix formation—is illustrated in Figure 7.

The outcome of intra-node fusion is a set of local topological subgraphs, each representing the network structure inferred solely from the perspective of one seed node (node 1, 2, or 3). Although these subgraphs exhibit limited reconstruction accuracy when viewed in isolation, the fusion of multiple independent simulations per seed node plays a critical role: it mitigates the noise and uncertainty inherent in any single stochastic propagation path while simultaneously refining the evidential support for each potential edge. This process not only suppresses spurious correlations but also elevates the reliability of the local evidence. Consequently, the intra-node fusion step transforms raw, noisy simulation data into a coherent set of basic probability assignments (BPAs), thereby establishing a well-founded and consistent evidence base essential for the subsequent inter-node fusion stage.

#### 4.2.3. Second-Level Fusion: From Local Evidence to Global Consensus

Figure 8 visualizes the orthogonal fusion of the three evidence sets derived from nodes 1, 2, and 3 during the reconstruction of the Karate Club network. The fusion mechanism functions as an “information arbitrator and synthesizer,” dynamically integrating evidence from multiple local perspectives to yield a globally consistent topology. Specifically, for edges that lie entirely within a densely connected community, the existence belief is strongly reinforced through Dempster’s combination rule, as consistent support from multiple independent evidence sources leads to rapid belief accumulation. In contrast, for nodes located at community boundaries, the fusion mechanism explicitly addresses conflicting evidence from different observation perspectives: it quantifies the degree of conflict via the conflict coefficient *K* and redistributes belief masses according to the normalization factor 1/(1−K), thereby deriving a balanced affiliation decision.

A particularly notable outcome concerns bridge edges that connect distinct communities (e.g., the link between nodes 1 and 34). Although each single local perspective provides only weak and highly uncertain evidence for such inter-community connections, the fusion process extracts and combines spatially complementary observation clues. Through successive orthogonal combinations, belief supporting the existence of these bridging edges is progressively accumulated, eventually surpassing the decision threshold θ and enabling their correct recovery in the final reconstructed topology. Thus, the inter-node fusion stage not only consolidates locally consistent evidence but also resolves conflicts and uncovers structurally critical connections that are otherwise invisible from any single viewpoint.

The final globally reconstructed topology, shown in Figure 9a, exhibits structural completeness that substantially surpasses any single-perspective reconstruction. A detailed comparison with the ground-truth network reveals notable achievements at two levels: At the macroscopic topological level, the method accurately recovers the two characteristic community structures centered around nodes 1 and 34. It not only correctly identifies core members within each community but also clearly delineates the boundaries between them. At the microscopic connection level, the approach successfully identifies key bridge edges linking the two communities, including weak ties that are difficult to infer from partial observations. These reconstruction results align closely with the known social context: the karate club eventually split into two factions, one led by the instructor (node 1) and the other by the administrator (node 34). Importantly, the reconstructed bridge connections correspond to individuals who attempted to mediate the real-world conflict. This outcome demonstrates that the two-level fusion framework can effectively integrate fragmented information from multiple observation perspectives. By progressively combining supportive evidence and resolving conflicting information, the framework ultimately reconstructs a global topology that closely mirrors the actual social structure, highlighting the method’s strong reconstruction capability.

#### 4.2.4. Performance Comparison and Discussion

After comparing the method in this article (TBFF) with a network reconstruction method (recalled NRA) based on discrete state sequences [46], as shown in Figure 9b, the reconstruction result nodes of NRA contain a large number of redundant edges, which blur the community structure that the original network should have. The NRA method infers edges by counting co-infection events: $n_{ij}=\sum_{t}I\left(X_{it}=1\wedge X_{jt}=1\right)$, where *X* is the binary infection matrix. This simple counting approach cannot distinguish direct transmissions from indirect co-infections. Compared with the true topology in Figure 9a, the proposed method not only accurately restores the community division centered on nodes 1 and 34 but also recovers inter-node connections with high fidelity, yielding a reconstructed topology that is visually highly consistent with the ground truth. Figure 9c,d further show that while both methods achieve high true positive rates (TPR) in detecting actual edges, the reconstruction produced by our method is considerably more reliable. The NRA method yields a false positive rate (FPR) nearly equal to its TPR in value, indicating that a large proportion of its inferred edges are spurious. In contrast, our method maintains a consistently low FPR throughout the reconstruction. Specifically, for the Karate Club network, the TBFF attains a TPR of 0.987 while keeping the FPR as low as 0.022. Under the same setting, the NRA method reaches a similar TPR of 0.987, but its FPR rises to 0.966, reflecting a substantial number of false positives.

This comparison indicates that the NRA method suffers from over-inference: its higher true positive rate comes at the cost of a substantially elevated false positive rate. In contrast, the two-level belief fusion framework (TBFF) introduced in this paper processes uncertain and conflicting information through two successive applications of D-S evidence theory. This design strictly suppresses the generation of spurious edges while maintaining a high true positive rate. The detailed analysis of this real-world case demonstrates that the core strength of TBFF lies not merely in recovering existing connections but also in the overall accuracy and reliability of the reconstructed topology. By effectively extracting informative evidence and filtering out non-existent links, the framework produces a network structure that is both topologically clear and faithfully representative of the underlying system.

### 4.3. Large-Scale Real-World Network Reconstruction Results

Following the detailed case analysis of the Karate Club network, this section extends the evaluation to four additional large-scale real-world networks: American Airlines, German Highway, Copenhagen Twitter Forwarding, and Yeast Protein–Protein Interaction (PPI). These networks span diverse domains, sizes, and edge densities, providing a comprehensive testbed to assess the generalizability of the proposed Two-level Belief Fusion Framework (TBFF). Table 1 summarizes the true positive rate (TPR) and false positive rate (FPR) achieved by TBFF and the baseline Network Reconstruction Algorithm (NRA) across all five networks, offering a quantitative performance comparison.

The comparative analysis of the results in the table demonstrates that the method proposed in this paper (TBFF) possesses the following advantages over the baseline NRA approach.

Reliability of the reconstructed information. Across all real networks, the false positive rate (FPR) of TBFF remains significantly lower than that of NRA. For example, in the Copenhagen Twitter forwarding network, TBFF achieves an FPR of only 0.012, whereas NRA yields an FPR as high as 0.9999, indicating that nearly all edges inferred by NRA are incorrect. Similarly, in the German highway network, TBFF maintains an FPR of 0.022, while NRA reaches 0.500. These outcomes highlight TBFF’s superior ability to handle conflicting and uncertain information, thereby generating more reliable network topologies.

Precision in reconstructing actual connections. In terms of the true positive rate (TPR), TBFF accurately recovers most existing edges across all five real networks. In the Karate Club network and the German highway network, TBFF achieves TPR values of 0.987 and 0.983, respectively, substantially outperforming the NRA method in reconstruction accuracy.

Generalizability and adaptability of the framework. TBFF maintains high performance across networks of varying scales and types, from a small social network with 34 nodes to a large-scale biological network with 2375 nodes. Notably, even on large-scale sparse networks such as the German highway network, TBFF achieves a TPR of 0.983, demonstrating its robustness and adaptability to diverse network structures.

**Figure 10:**
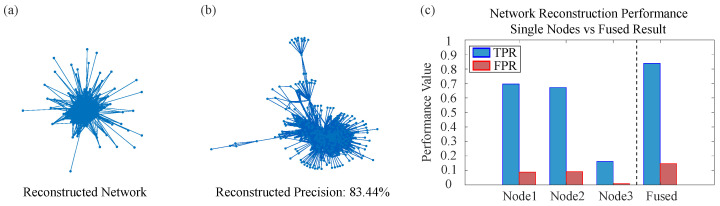
Network Reconstruction Results for US Airline Network. (**a**) Reconstructed topological structure of the US Airline network, (**b**) Reconstruction accuracy visualization mapping the precision of edge recovery, (**c**) TPR and FPR performance comparison.

**Figure 11:**
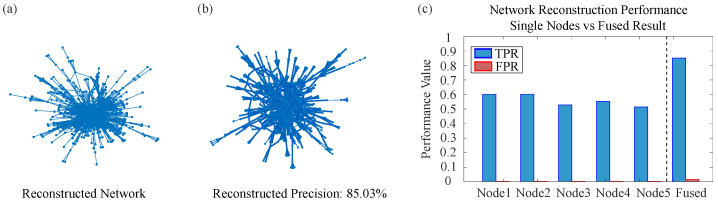
Network Reconstruction Results for the Copenhagen Twitter retweet network. (**a**) Reconstructed topological structure of the Copenhagen Twitter retweet network, (**b**) Reconstruction accuracy visualization mapping the precision of edge recovery, (**c**) TPR and FPR performance comparison.

**Figure 12:**
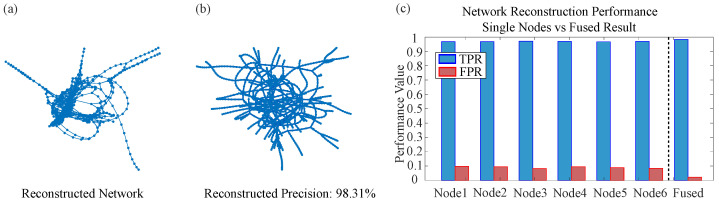
Network Reconstruction Results for German Railway Network. (**a**) Reconstructed topological structure of the German Railway network, (**b**) Reconstruction accuracy visualization mapping the precision of edge recovery, (**c**) TPR and FPR performance comparison.

**Figure 13:**
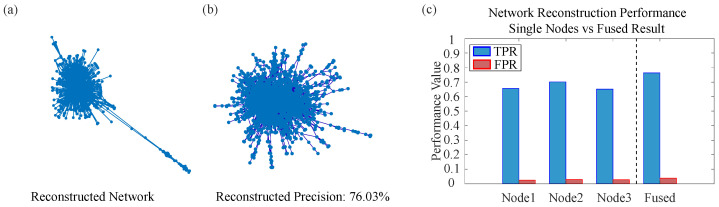
Network Reconstruction Results for Yeast PPI Network. (**a**) Reconstructed topological structure of the Yeast Protein–Protein Interaction network, (**b**) Reconstruction accuracy visualization mapping the precision of edge recovery, (**c**) TPR and FPR performance comparison.

Figure 10, Figure 11, Figure 12 and Figure 13 present the reconstruction results for the American Airlines network, the German Highway network, the Copenhagen Twitter forwarding network, and the Yeast PPI network, respectively. Each figure consists of three panels: the topology reconstructed by TBFF, a visualization of correctly recovered edges, and a bar-chart comparison of TPR/FPR under single-node and multi-node fusion. These results confirm that, despite the scale and complexity of these networks, TBFF successfully recovers their core structural features and achieves excellent reconstruction metrics. Overall, the two-level belief fusion framework shows strong practical value in addressing real-world complex network reconstruction problems.

The systematic experiments conducted on large-scale real-world networks demonstrate that the proposed two-level belief fusion framework consistently surpasses the baseline method in terms of reliability, scalability, and generalization. These outcomes confirm the framework’s effectiveness and practical value in addressing real-world network reconstruction challenges, especially in scenarios that require highly accurate and trustworthy reconstruction results.

### 4.4. Discussion

This chapter comprehensively evaluates the proposed two-level belief fusion framework through comparative reconstruction experiments on a randomly generated 16-node, 39-edge benchmark network and five real-world networks. The results consistently demonstrate that the framework achieves high precision, robustness, and versatility across all reconstruction tasks.

#### 4.4.1. Methodological Foundations of Performance Advantages

The primary advantage of the framework lies in its hierarchical processing of uncertain information, which effectively addresses conflicting evidence and the substantial uncertainty inherent in observational data.

First, intra-node fusion serves as the first-level processing stage, mitigating the considerable random uncertainty present in the initial data. From any single-node observation perspective, the stochasticity of individual SIR propagation simulations introduces significant noise into the evidence captured in the association matrix. As illustrated in Section 4.2.1, this stage applies multiple independent simulations coupled with D-S combination to filter out uncontrollable uncertainties. This process enhances the quality of evidence from a single perspective, yielding reliable Basic Probability Assignments (BPAs) that form a solid foundation for subsequent inter-node fusion.

Second, inter-node fusion constitutes the second-level processing, designed to overcome the inherent limitations of any locally obtained evidence. As vividly demonstrated in Section 4.2.1, evidence from a single node is fundamentally partial. The strength of the inter-node fusion step lies in its powerful information integration capability. Within the rigorous mathematical framework of D-S evidence theory, evidence from different observational perspectives is sequentially and orthogonally combined. This process enables the reconstruction results to evolve from locally optimal evidence to a globally superior topology.

In summary, the success of the proposed method stems from its rigorous two-level belief fusion design: the first level improves single-source evidence quality, while the second level synthesizes multi-source perspectives for further refinement. This hierarchical mechanism for handling uncertainty and conflict is the cornerstone of the method’s effectiveness.

#### 4.4.2. Limitations and Challenges

While the method demonstrates significant advantages, a rigorous scientific assessment must also acknowledge its current limitations, which point toward valuable future research directions.

Regarding computational complexity, the main cost arises from the large-scale SIR simulations required for initial data generation and the subsequent two-level evidence fusion calculations. Although the $O(N^{2})$ time and space complexity is manageable for networks with thousands of nodes, it remains a challenge for ultra-large-scale networks comprising millions of nodes.

In terms of parameter configuration, the reconstruction performance of the method depends to some degree on how well the SIR simulation and evidence decision parameters align with the target network’s characteristics. While this study demonstrates the method’s potential through case-specific parameter tuning, developing an adaptive parameter configuration strategy for scenarios with completely unknown prior information remains an open problem.

A further consideration is the optimal selection of seed nodes, as the final reconstruction result is inevitably influenced by this choice. Although the proposed two-level fusion mechanism exhibits some robustness to seed node selection, identifying the most informative seed nodes would maximize reconstruction accuracy while minimizing observational cost.

#### 4.4.3. Future Research Directions

Based on the above discussion, we outline several promising directions for future work:

- Algorithmic Optimization for Broader Applicability: We will explore sampling-based approximate algorithms to avoid exact computations over all $N^{2}$ node pairs, thereby extending the scale of networks that can be processed.
- Development of Adaptive Parameter Adjustment Mechanisms: Further investigation into the intrinsic relationship between dynamical model parameters and network characteristics will inform the development of adaptive configuration strategies, enhancing the method’s automation and generalizability.
- Intelligent Identification of Optimal Seed Nodes: Research will integrate graph information theory and active learning to design algorithms that actively select seed nodes that most effectively reduce initial data uncertainty, thereby implicitly optimizing the global evidence integration process.
- Extension of the Two-Level Fusion Framework: We plan to extend the framework to more challenging reconstruction problems, such as dynamic network reconstruction, edge weight inference in weighted networks, and even cross-layer prediction in multilayer networks, to further explore the potential of information fusion and evidence theory.
- Inference of Higher-Order and Mesoscopic Structures: The current framework focuses on pairwise interactions. A fundamental extension is to tackle systems with higher-order interactions, which require hypergraph reconstruction. This would involve adapting the propagation model and evidence formalism to hyperedges. Foundational work on hyperedge structural entropy [47] provides the necessary metrics to quantify complexity and uncertainty in such systems. Concurrently, incorporating mesoscopic structural priors—such as local clustering patterns quantified by clustering coefficient structural entropy [48]—could serve as valuable constraints to guide and refine the reconstruction of community-like structures within ordinary networks.

Through continued exploration of these directions, the two-level belief fusion framework has the potential to evolve into a powerful, general, and practical theoretical tool in network science.

## 5. Conclusions

Topological reconstruction of complex systems is a fundamental prerequisite for analyzing their operational principles. A persistent challenge in this endeavor is that interactions among system components are typically unknown and cannot be directly observed; thus, reconstructing the complete connectivity from a local observation perspective remains a key problem. Departing from traditional approaches, this paper innovatively integrates D-S evidence theory with information fusion, proposing a novel and rigorous two-level belief fusion framework that offers a complete solution for network reconstruction under local observation constraints.

The main contributions of this research are threefold:

At the methodological level, this work establishes a rigorous hierarchical belief fusion framework that systematically processes evidential uncertainty at two distinct stages. Intra-node fusion acts as the first filter, refining the stochastic uncertainty inherent in SIR propagation and purifying single-source evidence. Subsequently, inter-node fusion serves as the second filter, orthogonally integrating complementary or conflicting evidence from multiple sources, thereby overcoming the limitations of any single perspective and achieving a coherent synthesis of multi-source information. This layered, progressive reconstruction mode establishes a solid theoretical foundation for network reconstruction from partial observations.

At the algorithmic level, the paper provides a complete and computationally feasible reconstruction pipeline. Starting from SIR propagation simulations initiated from seed nodes, the workflow proceeds through time–state matrix aggregation, correlation matrix computation, two-level evidence fusion, and final threshold-based decision-making to generate the adjacency matrix. This end-to-end framework constitutes a systematic algorithm for inferring node connectivity.

At the experimental level, extensive evaluations on a benchmark network and multiple real-world networks demonstrate the framework’s superiority over traditional reconstruction methods reliant on discrete data. Our method exhibits two key capabilities: first, the ability to quantify evidential conflict and uncertainty; second, the capacity to holistically improve the quality of connectivity evidence by fusing information from multiple observational perspectives. The reconstruction results robustly validate the feasibility, accuracy, and practical applicability of the proposed framework for tackling real-world complex system reconstruction challenges.

This study demonstrates that applying D-S evidence theory to network reconstruction not only provides a powerful framework for handling uncertainty but also opens an innovative methodological pathway. Building upon this work, several promising directions merit further exploration: first, incorporating negative evidence (i.e., evidence against connections) to construct a bidirectional belief assessment framework, potentially reducing overall uncertainty at a fundamental level; second, extending the framework to accommodate dynamic and weighted network reconstruction; third, designing intelligent algorithms for optimal seed node selection to minimize observational cost while maximizing evidence quality. With continued investigation, the two-level belief fusion framework presented here is poised to become a more versatile and powerful tool in the network science repertoire. 

## Figures and Tables

**Table 1:** Comprehensive Comparison of Real-world Network Reconstruction Performance.

Network	Nodes	Edges	Density	TBFF (Ours)		NRA (Baseline)	
				TPR	FPR	TPR	FPR
Karate	34	78	0.139	0.987	0.022	0.987	0.966
US Airline	332	2126	0.037	0.801	0.119	0.974	0.997
CPH Twitter	761	1029	0.004	0.833	0.012	0.594	0.999
Ger Highway	1168	1243	0.002	0.983	0.022	0.346	0.500
Yeast PPI	2375	11,693	0.004	0.763	0.038	0.941	0.883

## Data Availability

The original contributions presented in this study are included in the article. Further inquiries can be directed to the corresponding authors.

## Appendix A. Step-by-Step Illustration of the Reconstruction Framework on a Benchmark Network

This appendix visually elaborates the methodological workflow outlined in Section 3.1, Section 3.2 and Section 3.3 using a concrete example. The following figures trace the reconstruction process for a specific node in a benchmark network, illustrating the key data transformations from simulation to final decision.

## References

[1] Jereesh A.S., Kumar G.S. (2024). Reconstruction of gene regulatory networks using graph neural networks. Appl. Soft Comput..

[2] Yuan Q., Duren Z. (2025). Inferring gene regulatory networks from single-cell multiome data using atlas-scale external data. Nat. Biotechnol..

[3] Zhang Y., Zhang Y., Zhao Y., Deng S., Yang Y. (2024). Dual variational graph reconstruction learning for social recommendation. IEEE Trans. Knowl. Data Eng..

[4] Liu J.-B., Zheng Y.-Q., Lee C.-C. (2024). Statistical analysis of the regional air quality index of yangtze river delta based on complex network theory. Appl. Energy.

[5] Muthuvel D., Sivakumar B. (2024). Spatial propagation of different drought types and their concurrent societal risks: A complex networks-based analysis. J. Hydrol..

[6] Peel L., Peixoto T.P., De Domenico M. (2022). Statistical inference links data and theory in network science. Nat. Commun..

[7] Peixoto T.P. (2018). Reconstructing networks with unknown and heterogeneous errors. Phys. Rev. X.

[8] Peixoto T.P. (2019). Network reconstruction and community detection from dynamics. Phys. Rev. Lett..

[9] Peixoto T.P. (2025). Uncertainty quantification and posterior sampling for network reconstruction. Proc. R. Soc. A.

[10] Peixoto T.P. (2025). Network reconstruction via the minimum description length principle. Phys. Rev. X.

[11] Shore J., Johnson R. (2003). Axiomatic derivation of the principle of maximum entropy and the principle of minimum cross-entropy. IEEE Trans. Inf. Theory.

[12] Stuart J.M., Segal E., Koller D., Kim S.K. (2003). A gene-coexpression network for global discovery of conserved genetic modules. Science.

[13] Zhang Z., Chen Y., Mi Y., Hu G. (2019). Reconstruction of dynamic networks with time-delayed interactions in the presence of fast-varying noises. Phys. Rev. E.

[14] Donges J.F., Zou Y., Marwan N., Kurths J. (2009). The backbone of the climate network. Europhys. Lett..

[15] Zhou D., Xiao Y., Zhang Y., Xu Z., Cai D. (2013). Causal and structural connectivity of pulse-coupled nonlinear networks. Phys. Rev. Lett..

[16] Runge J., Bathiany S., Bollt E., Camps-Valls G., Coumou D., Deyle E., Glymour C., Kretschmer M., Mahecha M.D., Muñoz-Marí J. (2019). Inferring causation from time series in earth system sciences. Nat. Commun..

[17] Casadiego J., Nitzan M., Hallerberg S., Timme M. (2017). Model-free inference of direct network interactions from nonlinear collective dynamics. Nat. Commun..

[18] Levnajić Z. (2012). Dynamical networks reconstructed from time series. arXiv.

[19] Wang W.-X., Lai Y.-C., Grebogi C. (2016). Data based identification and prediction of nonlinear and complex dynamical systems. Phys. Rep..

[20] Han X., Shen Z., Wang W.X., Di Z. (2015). Robust reconstruction of complex networks from sparse data. Phys. Rev. Lett..

[21] Wang W.-X., Yang R., Lai Y.-C., Kovanis V., Harrison M.A.F. (2011). Time-series–based prediction of complex oscillator networks via compressive sensing. Europhys. Lett..

[22] Buntine W. (2002). A guide to the literature on learning probabilistic networks from data. IEEE Trans. Knowl. Data Eng..

[23] Runge J. (2018). Causal network reconstruction from time series: From theoretical assumptions to practical estimation. Chaos Interdiscip. J. Nonlinear Sci..

[24] Liu H., Kim J., Shlizerman E. (2018). Functional connectomics from neural dynamics: Probabilistic graphical models for neuronal network of caenorhabditis elegans. Philos. Trans. R. Soc. B Biol. Sci..

[25] Ma C., Chen H.-S., Li X., Lai Y.-C., Zhang H.-F. (2020). Data based reconstruction of duplex networks. SIAM J. Appl. Dyn. Syst..

[26] Zhang Z., Zhao Y., Liu J., Wang S., Tao R., Xin R., Zhang J. (2019). A general deep learning framework for network reconstruction and dynamics learning. Appl. Netw. Sci..

[27] Ma C., Chen H.-S., Lai Y.-C., Zhang H.-F. (2018). Statistical inference approach to structural reconstruction of complex networks from binary time series. Phys. Rev. E.

[28] Chen M., Zhang Y., Zhang Z., Du L., Wang S., Zhang J. (2022). Inferring network structure with unobservable nodes from time series data. Chaos An Interdiscip. J. Nonlinear Sci..

[29] Xiang B.-B., Ma C., Chen H.-S., Zhang H.-F. (2018). Reconstructing signed networks via ising dynamics. Chaos Interdiscip. J. Nonlinear Sci..

[30] Zhang Y., Guo Y., Zhang Z., Chen M., Wang S., Zhang J. (2022). Universal framework for reconstructing complex networks and node dynamics from discrete or continuous dynamics data. Phys. Rev. E.

[31] Zhan T., Li Z., Deng Y. (2024). Random graph set and evidence pattern reasoning model. arXiv.

[32] Li M., Li L., Zhang Q. (2024). Information fusion and decision-making utilizing additional permutation information. Mathematics.

[33] Tang Y., Wu K., Li R., Guan H., Zhou D., Huang Y. (2025). Probabilistic transformation of basic probability assignment based on weighted visibility graph networks. Appl. Soft Comput..

[34] Li M., Zhang Q. (2025). Local entropy and nonextensivity of networks ensemble. Commun. Nonlinear Sci. Numer. Simul..

[35] Zachary W.W. (1977). An information flow model for conflict and fission in small groups. J. Anthropol. Res..

[36] Kermack W.O., McKendrick A.G. (1927). A contribution to the mathematical theory of epidemics. Proc. R. Soc. Lond. Ser. A Contain. Pap. Math. Phys. Character.

[37] Deng Y. (2015). Generalized evidence theory. Appl. Intell..

[38] Li M., Li L., Zhang Q. (2024). A new distance measure between two basic probability assignments based on penalty coefficient. Inf. Sci..

[39] Deng X., Jiang W. (2025). Upper bounds of uncertainty for Dempster combination rule-based evidence fusion systems. IEEE Trans. Syst., Man Cybern. Syst..

[40] Dempster A.P. (2008). Upper and lower probabilities induced by a multivalued mapping. Classic Works of the Dempster-Shafer Theory of Belief Functions.

[41] Li M., Zhang Q., Deng Y. (2018). Evidential identification of influential nodes in network of networks. Chaos Solitons Fractals.

[42] Friedrich M. (2017). Functional structuring of road networks. Transp. Res. Procedia.

[43] Rossi R., Ahmed N. (2014). Role discovery in networks. IEEE Trans. Knowl. Data Eng..

[44] Rossi R.A., Ahmed N.K. (2015). The network data repository with interactive graph analytics and visualization. Proceedings of the AAAI Conference on Artificial Intelligence, Austin, TX, USA, 25–30 January 2015.

[45] Colizza V., Pastor-Satorras R., Vespignani A. (2007). Reaction–diffusion processes and metapopulation models in heterogeneous networks. Nat. Phys..

[46] Xu X., Zhu C., Zhu X.-Q. (2021). Discrete data based local-to-global network reconstruction algorithm. Acta Phys. Sin..

[47] Xian Y., Chen L., Li M., Zhang Q. (2025). Topological complexity quantification in hypergraphs networks via hyperedge-based entropic measures. Phys. Lett. A.

[48] Zhang Z., Li M., Zhang Q. (2024). A clustering coefficient structural entropy of complex networks. Phys. A Stat. Mech. Its Appl..

